# Polysaccharides Extracted from *Rhizoma Pleionis* Have Antitumor Properties In Vitro and in an H22 Mouse Hepatoma Ascites Model In Vivo

**DOI:** 10.3390/ijms19051386

**Published:** 2018-05-07

**Authors:** Yukun Fang, Anhong Ning, Sha Li, Shaozheng Zhou, Lei Liu, Thomson Patrick Joseph, Mintao Zhong, Jilong Jiao, Wei Zhang, Yonghui Shi, Meishan Zhang, Min Huang

**Affiliations:** Department of Microbiology, Dalian Medical University, Dalian 116044, China; fangyukun90@163.com (Y.F.); yynah-64@163.com (A.N.); dlshali@dmu.edu.cn (S.L.); zhoushaozheng1@126.com (S.Z.); liulei875@163.com (L.L.); microbiologist02@gmail.com (T.P.J.); zhmt251a@163.com (M.Z.); jiaoem@sina.cn (J.J.); zhangw@dlmedu.edu.cn (W.Z.); soniasyh@163.com (Y.S.); 18940876833@163.com (M.Z.)

**Keywords:** *Rhizoma Pleionis*, orchidaceae, malignant ascites, polysaccharides, H22 hepatoma cells

## Abstract

Malignant ascites is a highly severe and intractable complication of advanced or recurrent malignant tumors that is often immunotherapy-resistant. *Rhizoma Pleionis* is widely used in traditional medicine as an antimicrobial and anticancer agent, but its effectiveness in treating malignant ascites is unclear. In the current study, we investigated the effect of polysaccharides isolated from *Rhizoma Pleionis* (PRP) on murine hepatocarcinoma H22 cells in an ascites model. We have found that the main components of PRP, that presented a relative molecular weight of 383.57 kDa, were mannose and glucose. We also found that PRP reduced the occurrence of abdominal ascites and increased survival in our mouse model. An immune response in the ascites tumor model was observed by performing a lymphocytes proliferation experiment and an E-rosette test. The ratios of CD8+ cytotoxic T cells and NK cells in the spleen were examined by flow cytometry, and the mRNA expression of Foxp3+in CD4^+^CD25^+^ (T regulatory Tregs) was measured by RT-PCR (reverse transcription-polymerase chain reaction). The levels of the cytokines TNF-α (tumor necrosis factor), VEGF (vascular endothelial growth factor), IL-2 (interleukin), and IFN-γ (interferon) in the serum and ascites supernatants were measured by ELISA. The expression of Foxp3 and Stat3 in peritoneal cells in the mouse model was measured by immunocytochemistry. The results indicated that PRP increased H22 tumor cell apoptosis in vivo by activating and enhancing the immune response. Furthermore, the effects of PRP on the proliferation of H22 cells were assessed by the CCK8 assay, Hoechest 33258, and TUNEL staining in vitro. We found that PRP suppressed the proliferation of H22 tumor cells but had no effect on BRL (Big rat liver) -3A rat hepatoma normal cells in vitro. Next, we investigated the underlying immunological mechanism by which PRP inhibits malignant ascites. PRP induced tumor cell apoptosis by inhibiting the Jak1–Stat3 pathway and by activating Caspase-3 and Caspase-8 to increase the Bax/Bcl-2 ratio. Collectively, our results indicate that PRP exhibits significant antitumor properties in H22 cells in vivo and in vitro, indicating that PRP may be used as a new therapeutic drug for cancer treatment.

## 1. Introduction

Malignant ascites is caused by cancer-associated accumulation of fluids in the peritoneal cavity. Approximately 50% of patients with advanced or recurrent tumors develop malignant ascites [1,2]. The most frequent neoplasms associated with ascites include hepatocellular, ovarian, and gastric carcinomas, as well as pancreatic adenocarcinomas [2,3]. Malignant ascites is often a sign of advanced disease and poor prognosis in many malignancies [4,5]. There are no effective approaches for treating malignant ascites, and therefore it is considered one of the most serious complications in cancer patients, as well as one of the major causes of cancer-related mortality. At present, chemotherapy, abdominal paracentesis, diuretics, and peritoneovenous shunting are widely used treatments to relieve ascites symptoms [6,7,8,9]. However, these methods only relieve the symptoms and do not resolve the underlying problem. Moreover, these therapies have many side effects, such as peritoneal adhesion, peritonitis, hypovolemia, and protein loss [10,11]. Unfortunately, the high prevalence of metastasis and ascites is often associated with poor prognosis in patients in advanced stages of cancer [12]. Therefore, finding more effective treatments for malignant ascites has become a primary focus of the current pharmaceutical research.

Increasing evidence shows that traditional Chinese medicine (TCM) could offer potential approaches for treating malignant ascites [13]. It has been shown that TCM can alleviate the toxic effects of antitumor therapies in some tumor patients [14]. For example, natural polysaccharides, such as lentinan, have been used to treat hepatocellular carcinoma [15,16]. *Rhizoma Pleionis* (Orchidaceae) (RP) is a pseudobulb of orchid plants (*Cremastra appendiculata*) that has been used to reduce heat and counteract toxins in TCM. In addition, RP has a variety of other bioactive properties [17]. RP extracts can effectively inhibit the proliferation of mouse breast cancer cells and human H29T cells [18,19]. RP is often used concomitantly with other TCMs, such as Lufengfang, which has been shown to inhibit the invasive properties of MDA-MB-231 cells. The underlying mechanism of RP might involve decreasing the matrix metalloproteinase-9 (MMP-9) mRNA/tissue inhibitor of metalloproteinase-1 (TIMP-1) mRNA ratio [20].

The aim of this study was to purify the polysaccharide component of RP (PRP) and investigate its possible anti-ascites properties. Furthermore, we established a mouse xenograft model of ascites to study the underlying mechanism of PRP’s anti-ascites effects in vitro and in vivo.

## 2. Results

### 2.1. PRP Analysis 

**Figure 1:**
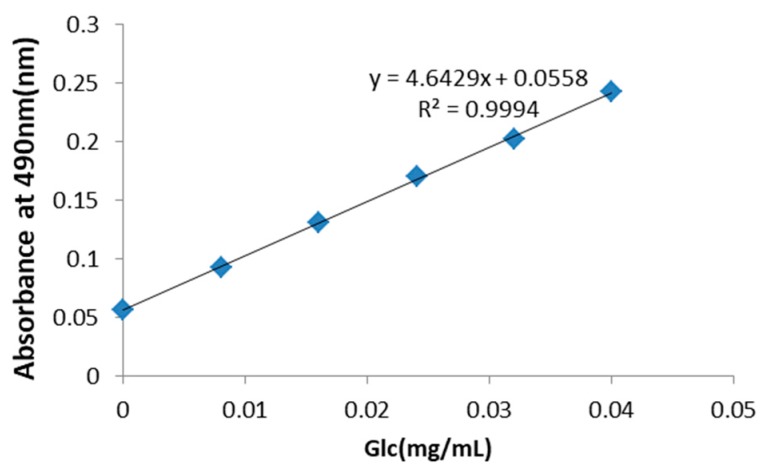
Standard curve for the determination of the total concentration of polysaccharides in *Rhizoma Pleionis* (PRP). The total saccharide concentration in PRP was determined to be 30 mg/mL using d-glucose as a standard.

In this study, we isolated PRP by hot water extraction and ethanol precipitation. The polysaccharides were purified, and their structure and relative molecular mass were determined. As shown in Figure 1, the concentration of total saccharides in PRP was 30 mg/mL, as established using d-glucose as a standard. The main water-soluble polysaccharides isolated from RP were mannose (6067.76 μg/mL) and glucose (3744.62 μg/mL), followed by galactose (60.82 μg/mL) and arabinose (50.50 μg/mL), as well as xylose (21.99 μg/mL), fructose (8 μg/mL), and rhamnose (4.34 μg/mL) in small amounts (Table 1). The molecular weight of PRP was 383.57 kDa (Table 2). CD-spectrum circular dichroism spectrum) (analysis demonstrated that the secondary structure of PRP consisted of an anti-parallel and random coil at 180–260 nm, followed by a beta-turn, and a helix in parallel (Table 3).

### 2.2. Antitumor Effects of PRP In Vivo

**Figure 2:**
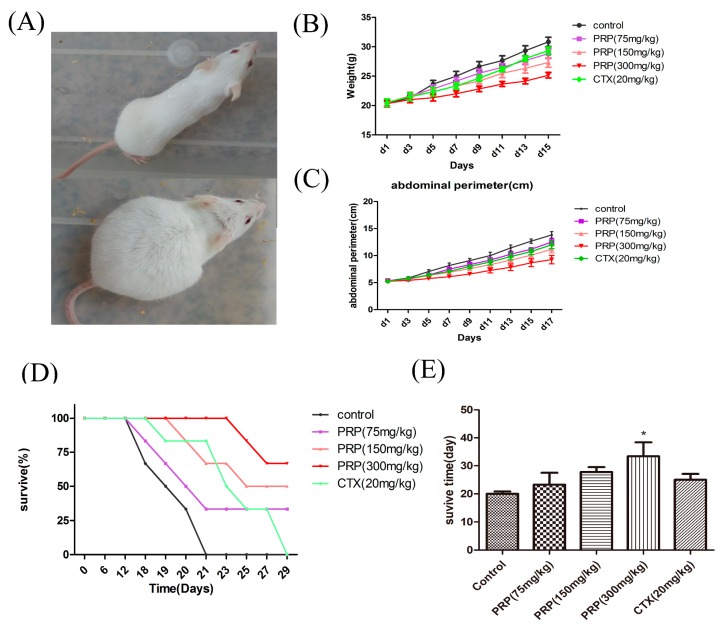
Effects of PRP treatment on the H22 malignant ascites model (Control group, PRP treatment groups, Cyclophosphamide(CTX) group). (**A**) H22 malignant ascites mouse model. (**B**) Abdominal perimeter of mice bearing H22 ascites tumors in five different experimental groups. (**C**) Mean mouse weight in each group. (**D**) Mice survival curve in each group. (**E**) Survival rate of each group by the 50th day. The data are presented as the mean ± SD (*n* = 8). (* *p* < 0.05).

To investigate if PRP suppresses H22 tumor growth in vivo, we established a malignant ascites model in BALB/c mice (Figure 2A). We transplanted H22 cells into the left abdominal cavity of mice and then treated the animals with PRP in saline at different doses (75, 150, and 300 mg/kg) or cyclophosphamide (CTX) (20 mg/kg). After five days, the control mice inoculated with tumor cells appeared to have an obvious abdominal bulge, while the mice treated with PRP presented no bulge. Seven days later, the ascites in the mice of the control group were increased, the bulge in the abdomen was prominent, the mice started facing difficulties in drinking and eating, became lazy, their hair was not as shiny, and their body weight and abdominal perimeter had rapidly increased. On the thirteenth day, the bulge in the control mice abdomen appeared like a ball, and the mice were apathetic and hardly active and began to die. We measured the abdominal perimeter and body weight following tumor inoculation from day 5 until day 15. The abdominal perimeter and body weight significantly decreased in the PRP-treated groups compared to the control group (Figure 2B,C). On the tenth day after tumor cell inoculation, the mouse abdominal perimeter and weight in the PRP treatment group were significantly smaller than those in the control group (Table 4 and Table 5). Furthermore, as shown in Figure 2D,E, the survival rate and survival time (by the end of the 50th day) increased in a dose-dependent manner in the PRP-treated group as compared to the control group. Collectively, these results demonstrate that PRP has significant antitumor properties in our H22 tumor model.

### 2.3. PRP-Mediated Effects on Cell Proliferation 

#### 2.3.1. Effects of PRP on T Cell and B Cell Spleen Lymphocyte Proliferation 

**Figure 3:**
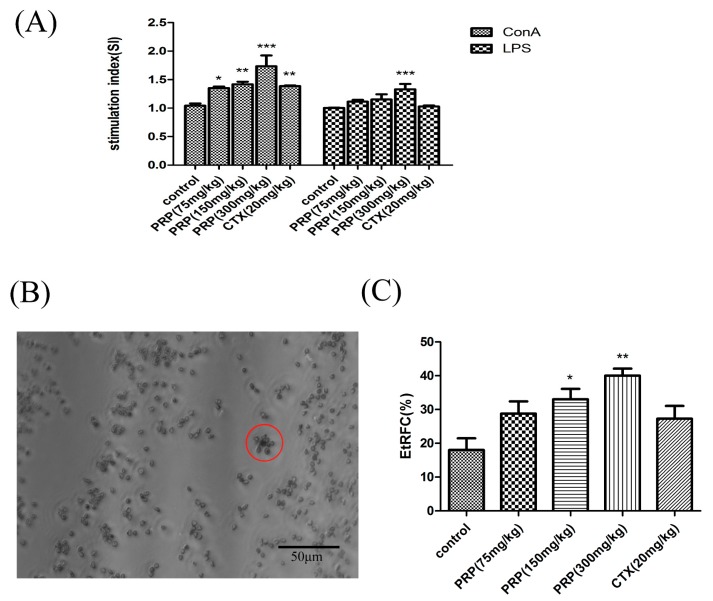
Effect of PRP treatment on T and B cell activation and proliferation. (**A**) Effects of PRP on spleen lymphocyte proliferation. (**B**,**C**) Garland structure of a sheep red blood cell with several T lymphocytes in red circle and the rate of E-rosette-forming cells (EtRFC) in each experimental group. The ratio of activation and proliferation was significantly higher in the treatment group compared to the control group. The data are shown as mean ± SD (*n* = 8). (* *p* < 0.05, ** *p* < 0.01, *** *p* < 0.001).

Next, we investigated if PRP affected splenic lymphocyte proliferation in our H22 tumor mouse model. We used concanavalin A (ConA) or lipopolysaccharide (LPS) to induce lymphocyte proliferation in H22 tumor-bearing mice in each group. The results show that the stimulation index of splenic lymphocyte proliferation dose-dependently increased with PRP treatment (Figure 3A). These data provide evidence that PRP increases splenic lymphocyte proliferation.

We then tested if the T cell CD2 receptor could specifically bind sheep red blood cells (SRBC) and form the characteristic garland (Figure 3B). Using this assay, we found that PRP increased the percentage of EtRFC (rate of E-rosette-forming cells) in a dose-dependent manner in the PRP-treated group compared to the control group (Figure 3C).

#### 2.3.2. Effects of PRP on CD8+ T Lymphocytes and NK Cells and Foxp3 mRNA Expression

**Figure 4:**
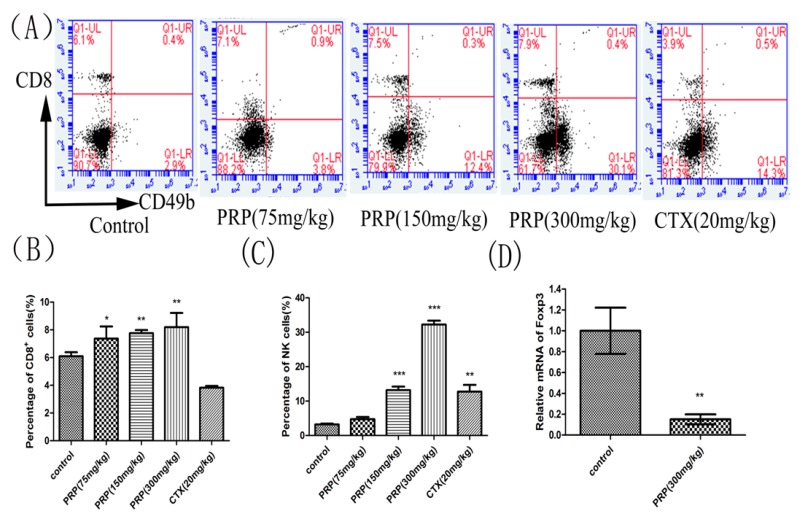
Effects of PRP treatment on spleen lymphocyte and Foxp3 mRNA expression. (**A**) Flow cytometry analysis of CD8+ T cells and CD49b+NK cells in different groups. (**B**,**C**) Effects of PRP on the percentage of splenic CD8+ T lymphocytes and NK cells. (**D**) Effects of PRP on spleen lymphocyte relative to Foxp3 mRNA expression. The data are presented as the mean ± SD (*n* = 8); (* *p* < 0.05, ** *p* < 0.01, *** *p* < 0.001) significantly different compared to the control group.

To further examine the role of PRP in antitumor immunity, we measured CD8^+^ and natural killer (NK) cell ratios in the spleen. Since Foxp3 is a key transcription factor that controls the development and function of T regulatory (Treg) cells, we measured Foxp3 mRNA expression in Tregs. As shown in Figure 4A–C, PRP the ratio of CD8^+^ T lymphocytes and NK cells increased dose-dependently in PRP-treated, H22 tumor-bearing mice compared to control mice by flow cytometry analysis. In contrast, the relative Foxp3 mRNA expression decreased in the PRP-treated group compared to the control group (Figure 4D). Given the results of the present study, we propose that PRP activates immune cells to facilitate an in vivo immunomodulatory effect in a H22 tumor model. Furthermore, we show that PRP treatment decreased Foxp3 expression in H22 malignant tumors, where it simulated Tregs and inhibited immune escape of tumor cells, reducing the development of malignant ascites.

#### 2.3.3. Foxp3 and Stat3 Immunocytochemical Analysis of Mouse Peritoneal Cells

**Figure 5:**
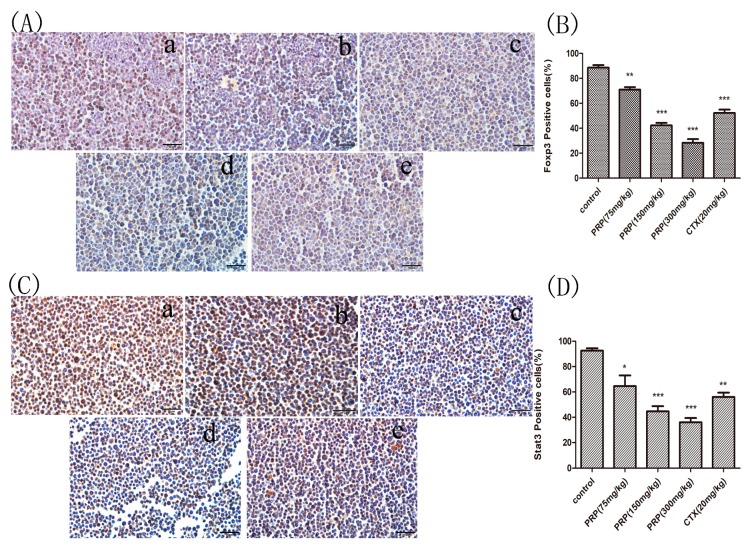
Foxp3 and Stat3 immunocytochemistry staining in ascites cells from mice. (**A**,**C**) Foxp3 and Stat3 immunocytochemistry images of ascites cells from mice in the various groups; (a) Control group; (b) low PRP treatment (75 mg/kg); (c) moderate PRP treatment (150 mg/kg); (d) high PRP treatment (300 mg/kg); (e) CTX (20 mg/kg). (**B**,**D**) The number of Foxp3- and Stat3-positive cells (moderate and strong brownish cytoplasmic staining) was calculated in five randomly selected areas in each experimental group as the number of positive cells/total compared to the control group. The data are presented as the mean ± SD (* *p* < 0.05, ** *p* < 0.01, *** *p* < 0.001) Original magnification, ×400.

Foxp3 and Stat3 activation serve as markers of disease recurrence. Therefore, we evaluated the expression of Foxp3 and Stat3 in peritoneal cells of the mouse model. As shown in Figure 5, fewer positive cells were observed in sections of PRP- and CTX-treated mice compared to the control group, and the number of Foxp3- and Stat3-positive cells dose-dependently decreased with PRP treatment. However, the expression of Foxp3 and Stat3 proteins in the PRP (150 and 300 mg/kg) groups was actually significantly lower than in the CTX group. Immunocytochemical analysis revealed that Foxp3 and Stat3 protein expression in the abdominal ascites were significantly reduced in PRP-treated tumor-bearing mice compared to control mice.

#### 2.3.4. PRP-Mediated Effects on Serum and Ascites Cytokine Levels

**Figure 6:**
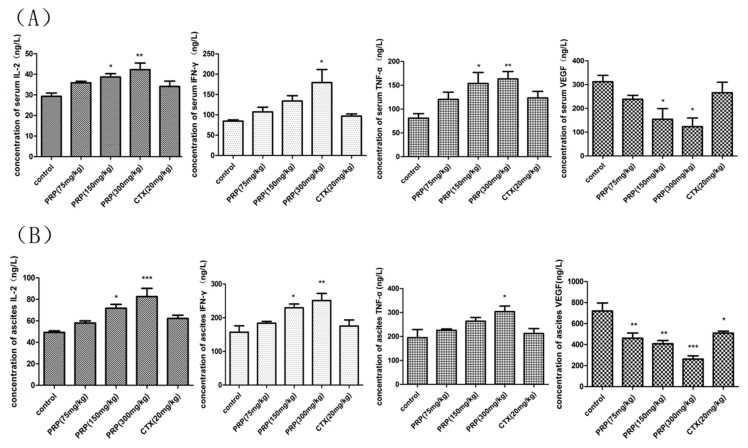
Effects of PRP on serum and ascites cytokines in different experimental groups. (**A**) Effects of PRP on serum cytokine levels in mice treated with different PRP concentrations or with CTX. (**B**) Effects of PRP on ascites cytokine levels in mice treated with different PRP concentrations or with CTX. The data are presented as the mean ± SD (*n* = 8); (* *p* < 0.05, ** *p* < 0.01, *** *p* < 0.001).

Cytokines are known to have both tumor-promoting and antitumor effects depending upon the specific context of the tumor microenvironment. As shown in Figure 6, interleukin-2 (IL-2), interferon-γ (IFN-γ), and tumor necrosis factor-α (TNF-α) levels dose-dependently increased in both serum and ascites from PRP-treated, H22 tumor-bearing mice compared to control mice. In contrast, vascular endothelial growth factor (VEGF) expression was reduced as the concentration of PRP increased. These findings indicate that PRP exerts its antitumor effects by stimulating the expression of different cytokines in vivo in a H22 mouse model.

#### 2.3.5. Mannose and Glucose Serum Concentrations 

**Figure 7:**
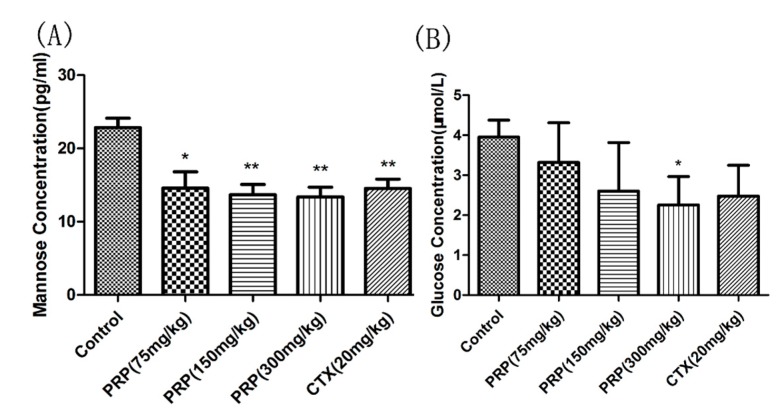
Mannose (**A**) and Glucose (**B**) serum concentrations. The data are means ± SD. (* *p* < 0.05, ** *p* < 0.01).

In order to determine the function of PRP in vivo, we measured mannose and glucose concentrations in mouse serum using ELISA. As shown in Figure 7, the concentrations of mannose and glucose decreased with PRP treatment. Moreover, their concentrations were much lower than those determined in in vitro experiments. These results suggest that the anticancer effect of PRP in vivo may be highly attributable to the induction of apoptosis.

### 2.4. PRP Antitumor Properties In Vitro

#### 2.4.1. Effect of PRP on H22 Tumor Cells and BRL-3A Rat Hepatoma Normal Cells In Vitro 

**Figure 8:**
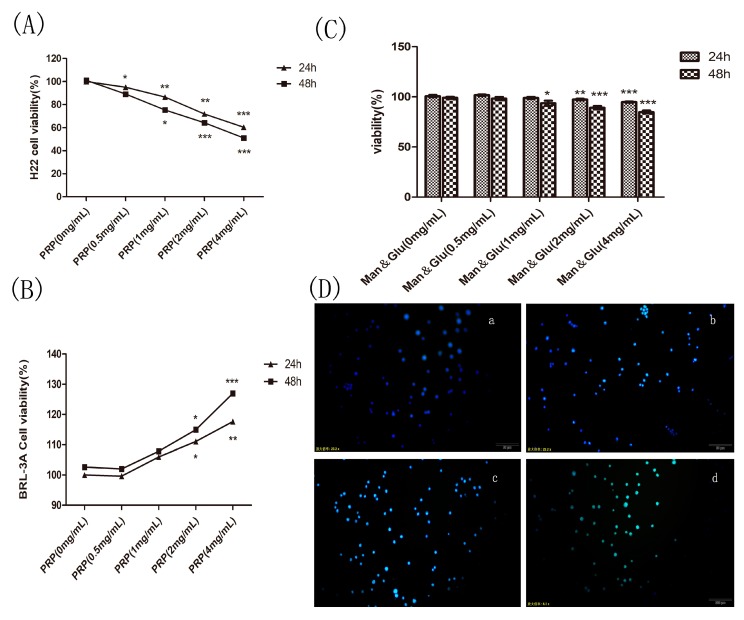
Effect of PRP and mannose and glucose (Man & Glu) on the proliferation of H22 cells and PRP toxicity test. (**A**,**B**) Effects of PRP on H22 cells and BRL-3A normal cell. (**C**) Effect of Man & Glu on the viability of H22 cells. (**D**) Fluorescence micrographs of H22 cells stained with Hoechst 33258. The cells were treated with (a) 0 mg/mL, (b) 1 mg/mL, (c) 2 mg/mL, and (d) 4 mg/mL PRP for 24 h (×400 magnification). The data are presented as the mean ± SD (* *p* < 0.05, ** *p* < 0.01, *** *p* < 0.001).

To investigate the possible toxicity of PRP, we measured cell viability using the CCK8 assay in vitro. H22 cells and BRL-3A rat hepatoma normal cells were cultured in vitro and treated with increasing concentrations of PRP for 24 or 48 h. As shown in Figure 8A,B, PRP treatment (4 mg/mL) resulted in 50% cell mortality in H22 cells after 48 h incubation but had no effect on BRL-3A cells.

#### 2.4.2. Effect of Mannose and Glucose on H22 Tumor Cells 

The main components of PRP are mannose and glucose. Therefore, to determine if these components affect H22 tumor cell proliferation, we treated cultured cells with mannose and glucose in a 6:4 solution at different concentrations (1, 2, and 4 mg/mL) for 24 or 48 h. As shown in Figure 8C, treatment with the low PRP concentration for 24 h, slightly increased cell proliferation, while the high concentration inhibited proliferation. After 48 h, the mixed solution inhibited H22 cell proliferation, but the maximum inhibition rate was no more than 16%, which was less than the effect of PRP on H22 cells at 48 h. These findings demonstrate that mannose and glucose do not interfere with PRP’s antitumor effect.

#### 2.4.3. PRP Induces Apoptosis in H22 Cells 

In the Hoechest33258 test, apoptotic H22 cells were marked by strong green fluorescence, and normal cells displayed weak fluorescence; necrotic cells were unstained. As shown in Figure 8D, PRP dose-dependently increased the number of apoptotic H22 cells. We also observed a reduction in the number of viable cells and other apoptotic features, such as fragmented chromatin.

**Figure 9:**
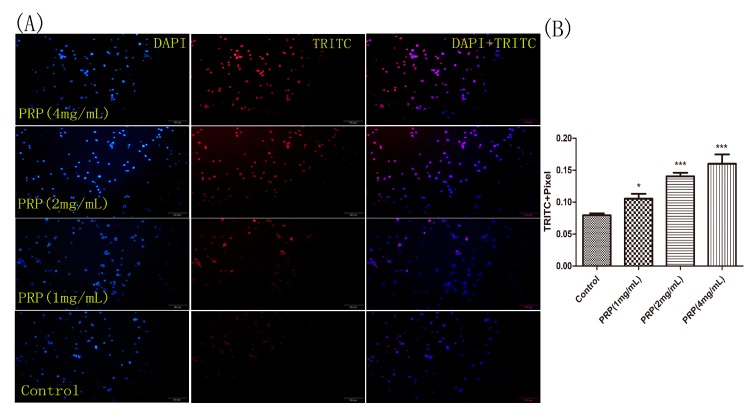
H22 cell staining for apoptosis using the TUNEL assay. (**A**) Representative images of cells stained with DAPI (left), TRITC (center), and merged (right). H22 cells were treated with different PRP concentrations for 24 h and then stained using the TUNEL assay. Apoptotic cells were examined by fluorescence microscopy. (×400 magnification) (**B**) The mean red fluorescence TRITC intensity of TUNEL-positive cells was analyzed by ImageJ software. The data are presented as mean ± SD. (*** *p* < 0.001, * *p* < 0.05).

We next measured PRP’s effects on cell apoptosis using Tunnel staining. Non-apoptotic cells were marked by blue nuclear fluorescence when stained with DAPI, and apoptotic H22 cells were marked by strong red fluorescence when stained with TRITC. As shown in Figure 9A, there was an increase in the number of TRITC-positive cells following PRP treatment compared to the control group. We performed a semi-quantitative analysis of TRITC intensity in five different regions of each group. The results also demonstrate that PRP treatment increased H22 cell apoptosis (Figure 9B) 

#### 2.4.4. Analysis of PRP’s Antitumor Activity In Vitro

Caspase-3 and Caspase-8 are important mediators of apoptosis. Therefore, we measured Caspase-3 and Caspase-8 enzyme activity using a caspase colorimetric assay in PRP-treated H22 cells. PRP significantly induced Caspase-3 and Caspase-8 activation in H22 cells compared to control untreated cells.

**Figure 10:**
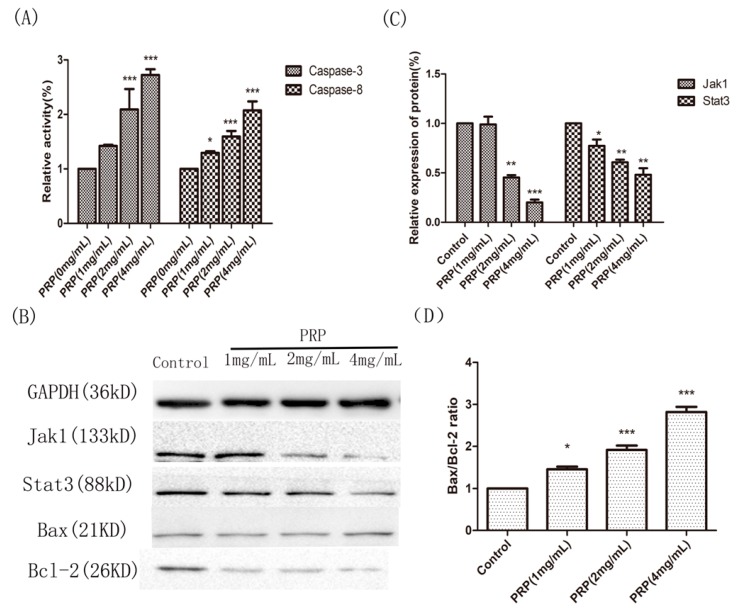
Effects of PRP treatment on the activation of Caspase-3 and Caspase-8 and on the expression of other related proteins. (**A**) Relative activities of Caspase-3 and Caspase-8 in H22 cells treated with PRP for 48 h. (**B**) Western-blot analysis of Jak1, Stat3, Bcl-2, and Bax expression (**C**,**D**) Relative quantative analysis ofJak1, Stat3, and the Bax/Bcl-2 ratio in each groups. The data are shown as mean ± SD; (* *p* < 0.05, ** *p* < 0.01, *** *p* < 0.01) significantly different from the control group.

To further define the underlying mechanisms of PRP’s antitumor activity, we measured Jak1, Stat3, Bcl-2, and Bax protein expression in vitro. Jak1, Stat3, and Bcl-2 protein levels were significantly upregulated, while Bax expression was only moderately increased in PRP-treated H22 cells compared to the untreated H22 cells. A quantitative analysis showed that PRP significantly inhibited the Jak1–Stat3 pathway and upregulated the ratio of Bax/Bcl-2. Collectively, these results show that PRP upregulates Bax and downregulates Bcl-2, thereby exerting antitumor properties through modulating the Jak1–Stat3 pathway (Figure 10).

## 3. Discussion

Several studies have reported that RP is an effective antitumor agent in TCM, but the underlying mechanism of its antitumor effects remains unknown [21]. In this study, we isolated PRP and determined its composition and structure. The major components of PRP were mannose and glucose, besides little amounts of probiotics like galactose, arabinose, xylose, fructose, and rhamnose, with a relative molecular weight of 383.57 kDa. We also investigated PRP’s antitumor effects in an in vivo ascites model using H22 tumor-bearing mice.

Our studies demonstrate that PRP can inhibit H22 tumor-associated ascites growth and metastasis in mice by improving immunity through the activation of the immune response and by inducing H22 cell apoptosis. On the basis of our in vivo experiments, we found that PRP exerted significant effects on the weight and abdominal perimeter in H22 tumor-bearing mice and also enhanced the survival of mice with malignant metastatic tumors. Furthermore, we showed that PRP significantly enhanced the antitumor immune response by stimulating T and B cell proliferation, increasing the CD8^+^/NK lymphocyte ratio, promoting secretion of IL-2, IFN-γ, and TNF-α, and decreasing VEGF expression. Moreover, PRP reduced Foxp3 and Stat3 expression in peritoneal cells from H22 tumor-bearing mice. In addition, PRP did not cause toxicity in BRL-3A rat hepatoma normal cells in vitro. Moreover, we excluded the influence of PRP’s main components, mannose and glucose, on H22 cells. Finally, we showed that PRP mediates its anti-proliferative effects by decreasing Jak1–Stat3 signaling and activating Caspase-8 and Caspase-3 signaling, with a subsequent increase in the Bax/Bcl-2 ratio.

The role of the immune system in cancer has been unappreciated for many decades because tumors effectively suppress immune responses by activating negative regulatory pathways that are associated with immune homeostasis or by adopting features that enable them to actively escape detection [22,23]. Therefore, a focus of tumor treatment strategies has been to improve the immunogenicity and sensitivity of effector cells to stimulate and enhance the antitumor immune response [24,25,26,27]. Our results indicate that PRP can be used as a novel immunomodulator to enhance the immune function by the non-specific activation of antitumor immune responses. We also measured mannose and glucose serum concentrations and found that mannose and glucose were lower in the treatment group compared to the control group. Moreover, their concentrations were much lower than in the in vitro experiments. This finding indicates that the anticancer effect of PRP in vivo is attributed not only to its direct effect on apoptosis but also to its ability to activate the immune response.

Unexpectedly, we found that the inhibitory effect of PRP on H22 cells in vitro is mediated by the inhibition of the Jak1–Stat3 signaling pathway, the activation of Caspase-3 and Caspase-8 signaling, and the increase in the Bax/Bcl-2 ratio. JAK/STAT proteins are critical regulators of cell growth, proliferation, differentiation, and immune regulatory functions [28,29]. Jak1 mutations were previously reported in various cancers, including leukemia, lung cancer, breast cancer, and hepatocellular carcinoma [30]. STAT3 plays an important signaling role within the cell, whereby it transmits signals from the outside of the cell to the nucleus [31,32,33]. In vitro studies have shown that Stat3 enhances the anti-apoptotic signaling through the induction of the Bcl-2 gene family, which can further influence the activation of caspase-induced apoptosis [34,35,36,37].

It is important to consider the potential side effects of new antitumor drugs. According to survey studies, repeated paracentesis is often the treatment of choice for malignant ascites, but its effects are limited [2]. Surgical removal of the tumor followed by combination chemotherapy is still the first choice in antitumor therapy, but chemotherapeutic drugs can destroy the immune system and allow the cancer to progress [38,39]. In this study, it can be observed that in the CTX group, the survival time of the mice decreased significantly as compared to that of the mice receiving the middle and high concentrations of PRP. The proliferation of lymphocytes, the regulation of the immune system, and the secretion of cytokines were also decreased with respect to the PRP-treated mice. Moreover, as an immunosuppressant, small dosage CTX can cause damage to the immune system, for example by the induction of lymphatic nuclear damage or by inhibiting lymphocyte proliferation [40,41]. However, we found that PRP exhibited antitumor properties with no toxicity to normal cells. In addition, PRP activated the immune system, amplified effector cell responses, and promoted the release of favorable cytokines.

## 4. Materials and Methods

### 4.1. Preparation of PRP

*Rhizoma Pleionis* (Baixin, Chinese Pharmaceutical Co. Ltd., Beijing, China) (100 g) was dried and powdered. RP was extracted three times using 1000 mL of water for 3 h at 100 °C. The whole extract was filtered and concentrated, followed by centrifugation. The supernatant was subjected to fractional precipitation with alcohol, as previously described [42]. Proteins in the crude PRP were removed using trichloroacetic acid, and the mixture was then washed in subsequent rounds of absolute ethyl alcohol, acetone, and diethyl ether [43,44]. Dialysis with pure water was performed, and PRP was further purified by DEAE (diethyl-aminoethanol) -52 cellulose ion exchange column chromatography. The concentration of total saccharides in PRP was determined using the sulfuric acid–phenol method and standardized to d-glucose [42]. The PRP secondary structure was identified by CD-spectroscopy, and the monosaccharide analysis was performed using ICP-MS (inductively coupled plasma massspectrometry).

### 4.2. In Vivo Study

#### 4.2.1. Animals and Tumor Formation

BALB/c mice (18 ± 2 g) were obtained from the Experimental Animal Centre at Dalian Medical University, China (Protocol number: AEE17015). (This experiment was approved by the ethics committee of Dalian Medical University in 12 July 2017). The animals were housed under standard specific-pathogen free (SPF) conditions (25 ± 1 °C and 55 ± 5% relative humidity) on a 12 h light/12 h dark cycle. The mice were fed a standard pellet diet, and water was supplied ad libitum. The animals were acclimated prior to experimentation for one week in quarantine.

The mouse H22 hepatocellular carcinoma cell line was purchased from ATCC and sub-cultured until the cells reached the logarithmic growth phase. Cells were then resuspended in saline at a concentration of 1 × 10^6^ cells/mL. BALB/c mice received 0.2 mL of the cell suspension (5 × 10^5^ cells per mouse) in the left peritoneal cavity, as previously described [45,46]. H22 tumor-bearing mice were randomly divided into five groups (*n* = 16 per group): cyclophosphamide group, in which the mice were treated with cyclophosphamide (20 mg/kg), control group, in which the mice were given the same volume of physiological saline, and the PRP-treated groups, in which the mice were treated with 75, 150, or 300 mg/kg PRP. All groups were administered treatment by intraperitoneal injection every other day, continuously for seven days. The abdominal perimeter and body weight of each mouse were measured daily. Half of the mice in every group were sacrificed at day 18, and the remaining mice were fed normally; their survival time was recorded.

#### 4.2.2. Splenic Lymphocyte Proliferation Assay

Using an aseptic technique, the splenocytes were isolated by disrupting the mouse spleen with a grinder in Mouse Lymphocyte Separation Medium (Darkewe, Shenzhen, China) and centrifuging at 800× *g* for 30 min. The cells were then washed and resuspended in RPMI-1640 medium (Gibco, Waltham, MA, USA) with 10% fetal bovine serum (FBS; TBD, Tianjin, China). Half of the lymphocytes were seeded in 96-well plates (1 × 10^6^/well) with concanavalin A (ConA, 2 mg/L) or lipopolysaccharide (LPS, 5 mg/L). The cells were further incubated for 48 h at 37 °C in a humidified atmosphere with 5% CO_2_. After 48 h, CCK8(Cell Counting Kit) (Biotool, Shanghai, China) was added to each well (20 μL/well), and the plates were incubated for additional 2 h, as previously described [47]. The absorbance of the splenic lymphocytes in each well was measured at 450 nm using an ELISA reader (Biotek, Winooski, VT, USA). The simulation index was calculated as follows: stimulation index (%) = absorbance value of the PRP-treated group/ absorbance value of the control group × 100%.

#### 4.2.3. Rate of Rosette-Forming Cell Test

The E receptors (CD2) on the surface of T cells bind to sugar peptides on the surface of sheep red blood cells (SRBC) (obtained from Senbeijia, Nanjing, China). When T cells are mixed with SRBC, the SRBC attach to the surface of the T cells, presenting a garland appearance, as previously described [48]. In brief, 0.1 mL of splenic lymphocyte suspension was mixed with 1% SRBC and 0.05 mL of inactivated FBS (TBD, Tianjin, China). The mixture was then centrifuged at low speed and incubated at 4 °C overnight. The next day, the cells were fixed with 0.8% glutaraldehyde on ice for 30 min, and the morphology of cytological smears was observed after Wright’s staining under a high-magnification microscope (Olympus, Tokyo, Japan). T cells that were able to bind three or more SRBCs were considered positive controls. Two hundred lymphocytes in five randomly selected areas were counted, and the percentage of garland formation was calculated.

#### 4.2.4. Flow Cytometry Analysis of Lymphocyte Subsets

Half of the isolated splenic lymphocytes were prepared for cultured using a Teflon pestle in RPMI-1640 medium. The sample was filtered, and the remaining erythrocytes were lysed. Single-cell suspensions of splenocytes (100 μL, 1 × 10^7^ cells/mL) were incubated with the following rat anti-mouse monoclonal antibodies: phycoerythrin (PE)-conjugated anti-CD8 and fluorescein isothiocyanate (FITC)-conjugated anti-CD49b (Abbkines, California, CA, USA) in the dark for 30 min at 4 °C, as previously described [49]. The percentage of CD8^+^ T cells and natural killer (NK) cells (indicated by CD49b+) were detected by flow cytometry (FACS Calibur, Becton Dickinson, Bergen, NJ, USA) and analyzed using Cell Quest software (Flowjo7.6, Stanford, CA, USA).

#### 4.2.5. Quantitative PCR (qPCR) Analysis of Foxp3 mRNA Expression

Lymphocytes were harvested and isolated from the spleens of PRP-treated (300 mg/kg) and control mice. For intracellular staining, monoclonal anti-CD4-PE and anti-CD25-APC antibodies (Abbkines) were used to stain surface markers. The percentages of CD4^+^CD25^−^ T cells and CD4^+^CD25^+^ T cells were calculated, and the cells were separated by flow cytometry (FACS Calibur, Becton Dickinson), as previously described [50]. Total RNA was isolated from CD4^+^CD25^+^ T cells using the RNAiso Plus kit (Solarbio, Beijing, China) according to the manufacturer’s protocol. RNA purity was assessed by spectrophotometry (Jinghua, Shanghai, China). Total RNA was reverse transcribed to cDNA using the TranstartGreen qPCR kit (TransGen Biotech, Beijing, China), and quantitative PCR was performed using Real-Time PCR System (ThermoFisher, Shanghai, China). The synthesized cDNA was used immediately for quantitative PCR (Bori, Hangzhou, China). To calculate differences in mRNA expression for each target, the 2^−ΔΔ*C*t^ method for relative mRNA quantitation was used with GAPDH gene expression as an endogenous control. The specific primers used were: Foxp3: 5′-AGTGCCTGTGTCCTCAATGGTC-3′ (forward); 5′-AGGGCCAGCATAGGTGCAAG-3′ (reverse); GAPDH: 5′-AAATGGTGAAGGTCGGTGTGAAC-3′ (forward); 5′-CAACAATCTCCACTTTGCCACTG-3′ (reverse).

#### 4.2.6. Serum and Ascites Cytokine Analyses 

Blood samples for cytokine analysis were collected from the mice eye orbits, and serum was separated. The ascites fluid was extracted and centrifuged, and the supernatant was collected. The levels of cytokine, including IL-2, IFN-γ, TNF-α, and VEGF, were measured by commercial ELISA kits (Langdun, Shanghai, China) according to the manufacturer’s instructions.

#### 4.2.7. Serum Mannose and Glucose Concentrations 

To determine the function of PRP’s main components in H22 tumor-bearing mice, we measured mannose and glucose concentrations in mouse serum using ELISA kits (Senbeijia, Nanjing, China), following the manufacturer’s instructions.

#### 4.2.8. Immunocytochemistry

After the ascites fluid was extracted by centrifugation, the sediment was washed twice in ice-cold phosphate buffered saline (PBS) and fixed with 4% paraformaldehyde for 24 h at 4 °C. Next, paraffin-embedded 4-μm-thick sections were cut for hematoxylin and eosin (H&E) staining and immunocytochemistry. All sections were deparaffinized using xylene and graded concentrations of ethanol in deionized water. Next, the sections were incubated in 10 mM citrate buffer (pH 6.0) at 90 °C for 20 min for antigen retrieval. The sections were then incubated overnight at 4 °C with an anti-Foxp3 (Cat No.: Abp53177-1) (Abbkines) or anti-Stat3 (Cat No.: 10253-2-Ap) (Proteinrech, Chicago, IL, USA) polyclonal antibodies at a 1:200 dilution and incubated with biotinylated goat anti-rabbit antibodies for 1 h at room temperature (ZSGB-Bio Kits (SP9000), Beijing, China). Then, the slides were washed with PBS and additionated with diaminobenzidine (DAB) chromogen for 3–5 min to yield a dark brown color. The slides were counterstained with hematoxylin and analyzed under a light microscope (Olympus, Tokyo, Japan).

Cells with moderate and strong brownish cytoplasmic staining were considered positive; unstained or weakly stained cells were considered negative, as previously described [50]. Positive cells were counted at 400× magnification in five randomly selected areas of each tumor sample, and the ratio of positive cells/total number of cells was calculated.

### 4.3. In Vitro Study

#### 4.3.1. Cell Culture and Viability Assay

H22 mouse hepatoma tumor cells and BRL-3A rat hepatoma normal cells were purchased from ATCC (Shanghai, China) and cultured in 1640 medium (Gibco, New York, NY, USA) supplemented with 10% fetal calf serum (TBD, Tianjin, China) in a humidified atmosphere (5% CO_2_, 37 °C). Next, 100 μL of cell suspension containing 1 × 10^4^ cells were added to each well of an E-plate 96 (Nest, Wuxi, China). Cells were then incubated overnight, and PRP or mannose and glucose at 0.5, 1, 2, or, 4 mg/mL were added to the wells in the E-plate 96 for 24 or 48 h. At the end of the experiment, the CCK-8 kit (Biotool, Shanghai, China) was used to measure cell viability, and absorbance was measured at 450 nm using a microplate reader (Biotek, Winooski, VT, USA).

#### 4.3.2. Nuclear Staining with Hoechst 33258

For Hoechst 33258, H22 cells (1 × 10^5^ cells/mL) were seeded in 6-well plates containing culture medium and different concentrations of PRP. After 24 h, the cells were collected by centrifugation and fixed with 4% paraformaldehyde for 10 min. The supernatant was removed, and 100 μL of Hoechst 33258 (KeyGen Biotech, Nanjing, China) was added to each well, followed by incubation at room temperature for 10 min. The stained cells were examined under a fluorescence microscope (Olympus, Tokyo, Japan).

#### 4.3.3. TUNEL Assay

For the TUNEL assay, we used a one-step TUNEL cell apoptosis detection kit (red TRITC-marked fluorescence detection method) (KeyGen Biotech) according to the manufacturer’s instructions. Briefly, H22 cells were treated with different concentrations of PRP for 24 h. Next, the cells were fixed with 4% paraformaldehyde for 30 min. After washing with PBS, 10% Proteinase K was added for 20 min, and then 50 μL of TdT was added for 1 h. After washing with PBS, we added 50 μL of Streptavavidin-TRITC and incubated the cells at 37 °C for 30 min. The nuclei were stained with DAPI (4′,6-diamidino-2-phenylindole), and the cells were observed using fluorescence microscopy (Olympus, Tokyo, Japan). The TRITC-positive cells were analyzed at 400× magnification in five randomly selected areas of each tumor sample using ImageJ software (Bethesda, Rockville, MD, USA).

#### 4.3.4. Caspase Activation Assay

To measure Caspase-3 and Caspase-8 activation, colorimetric assay kits (KeyGen Biotech) were used according to the manufacturer’s instructions. In brief, 3 × 10^6^ H22 cells were collected after treatment with different concentrations of PRP (1, 2, or 4 mg/mL) for 48 h. The wells were washed twice with PBS, and 50 μL of ice cold lysis buffer was added to the collected cells which were subsequently incubated on ice for 30 min. Next, the cell lysates were centrifuged at 10,000 rpm for 2 min at 4 °C, and the supernatants were collected and incubated with Reaction buffer/dithiothreitol (DTT) at 37 °C for 4 h with either a Caspase-3 or Caspase-8 substrates. After incubation, the absorbance was measured at 405 nm, using an ELISA microreader (Biotek, Winooski, VT, USA). Caspase-3 and Caspase-8 activities were calculated as relative activity (%) = absorbance value of the experimental sample/absorbance value of the control sample × 100%.

#### 4.3.5. Western Blot Analysis of Jak1, Stat3, Bax, and Bcl-2 

H22 cells were seeded at a density of 1 × 10^5^ cells/mL in 6-well plates (Biofil, Guangzhou, China) and incubated with different concentrations of PRP (1, 2, or 4 mg/mL) for 48 h. Next, the cells were washed twice in ice-cold PBS and harvested in RIPA buffer. Total proteins were quantified using a bicinchoninic acid (BCA) protein concentration assay reagent (KeyGen Biotech). The proteins (40 μg/μL) were then separated by 8% or 12% SDS/polyacrylamide gel electrophoresis (SDS/PAGE) and transferred to nitrocellulose membranes (Immobilon, Boston, MA, USA) at 200 mA for 1 h. The membranes were blocked with tris-buffered saline/Tween 20 (TBST), containing 5% skim milk at room temperature for 1 h. Next, the membranes were incubated overnight at 4 °C with primary polyclonal antibodies against Jak1, Stat3, Bcl-2, Bax, and GAPDH (Abbkines) at a 1:1000 dilution, as previously described [36]. The membranes were washed and incubated with a horseradish peroxidase-conjugated secondary anti-rabbit antibody (Abbkines) at a 1:1000 dilution for 1 h at room temperature. The membranes were finally washed, incubated in an ECL chemiluminescent detection kit (Proteintech, Chicago, IL, USA) according to the manufacturer’s instructions, and analyzed using the GelDoc 2000 system (Bio-Rad, Hercules, CA, USA). GAPDH was used to normalize protein loadings.

### 4.4. Statistical Analysis

SPSS 17.0 (IBM, Armonk, NJ, USA) software was used for the analyses. Prism 5 software (GraphPad5, San Diego, CA, USA) was used to create graphs. All experiments were repeated at least three times. The data are presented as means ± standard deviation (SD) and were analyzed using a two-tailed Student t-test or one-way analysis of variance (ANOVA) followed by Turkey tests for multiple comparisons.

## 5. Conclusions

In conclusion, we demonstrated that PRP has direct and indirect antitumor properties in a mouse model of ascites. PRP decreased proliferation of H22 cells in vitro and in vivo, as well as strengthened the immune system in our mouse model. Further studies are required to fully elucidate the underlying molecular mechanisms of PRP’s effects in human tumors.

## Figures and Tables

**Table 1:** PRP monosaccharide composition.

Monosaccharide Composition Analysis	Content (μg/mL)
Mannose	6067.76
Glucose	3744.62
Galactose	60.82
Arabinose	50.50
Fucose	21.99
Xylose	8.00
Rhamnose	4.34

**Table 2:** Molecular weight of PRP was determined to be 383.569.

Molecular	Weight (kDa)
Mn	39.877
Mw	383.569
Mz	2637
Mp	151.009
Mw/Mn	9.619

**Table 3:** Secondary structure of PRP as determined by circular dichroism spectrum.

Structures	180–260 nm	185–260 nm	190–260 nm	195–260 nm	200–260 nm	205–260 nm	210–260 nm
Helix	16.60%	16.30%	16.90%	14.70%	12.40%	11.60%	11.10%
Antiparallel	41.30%	44.30%	42.50%	30.00%	20.70%	17.40%	21.00%
Parallel	14.50%	14.10%	13.10%	16.50%	19.60%	22.00%	19.00%
Beta-Turn	21.50%	21.70%	20.80%	21.40%	22.50%	22.10%	23.20%
Rndm.Coil	44.00%	45.50%	43.30%	49.00%	54.20%	56.80%	55.70%
Total Sum	137.90%	142.00%	136.50%	131.60%	129.40%	129.90%	130.10%

**Table 4:** Mouse mean abdominal perimeter in each group.

Group (*n* = 8)	Abdominal (cm)			
	Day 1	Day 5	Day 10	Day 15
Control	5.33 ± 0.61	7.06 ± 0.81	9.33 ± 0.8	12.67 ± 0.98
PRP (75 mg/kg)	5.42 ± 0.58	6.42 ± 0.67	8.93 ± 0.79	11.17 ± 0.82
PRP (150 mg/kg)	5.30 ± 0.60	6.33 ± 0.52	8.13 ± 0.71	10.08 * ± 0.83
PRP (300 mg/kg)	5.41 ± 0.52	5.75 ± 0.41	7.25 ** ± 0.89	8.67 ** ± 1.75
CTX (20 mg/kg)	5.26 ± 0.61	6.41 ± 0.59	8.63 ± 0.67	10.75 ± 1.40

Compared with the control group (* *p* < 0.05, ** *p* < 0.01).

**Table 5:** Mouse mean weight in each group.

Group (*n* = 8)	Weight (g)			
	Day 1	Day 5	Day 10	Day 15
Control	20.3 ± 1.03	23.7 ± 1.50	26.7 ± 1.96	30.8 ± 1.94
PRP (75 mg/kg)	20.5 ± 1.51	22.8 ± 1.60	25.5 ± 1.64	28.8 ± 1.75
PRP (150 mg/kg)	20.5 ± 1.51	22.3 ± 1.21	24.2 ± 1.17	27.3 * ± 1.97
PRP (300 mg/kg)	20.3 ± 1.36	21.3 ± 1.36	22.8 * ± 1.03	25.2 ** ± 1.17
CTX (20 mg/kg)	20.5 ± 1.52	22.3 ± 1.75	24.7 ± 1.21	29.3 ± 1.50

Compared with the control group (* *p* < 0.05, ** *p* < 0.01).

## Abbreviations

PRP	*Rhizoma Pleionis* polysaccharides
TCM	Traditional Chinese medicine
SRBC	sheep red blood cells
EtRFC	Rate of E-rosette-forming cell
Man and Glu	Mannose and Glucose
TRITC	Tetramethylrhodamine
